# Intact high-level visual functions in congenital rod-monochromacy

**DOI:** 10.3389/fnins.2024.1418916

**Published:** 2024-09-27

**Authors:** Sheer Shabat, Ayelet McKyton, Deena Elul, Devora Marks Ohana, Einav Nahmany, Eyal Banin, Netta Levin

**Affiliations:** ^1^Department of Physical Medicine and Rehabilitation, Hadassah Medical Center and Faculty of Medicine, Hebrew University of Jerusalem, Jerusalem, Israel; ^2^fMRI Unit, Department of Neurology, Hadassah Medical Center and Faculty of Medicine, Hebrew University of Jerusalem, Jerusalem, Israel; ^3^Center for Retinal and Macular Degenerations (CRMD), Department of Ophthalmology, Hadassah Medical Organization and Faculty of Medicine, Hebrew University of Jerusalem, Jerusalem, Israel

**Keywords:** achromatopsia, faces, reading, scotopic, CNGA3, CNGB3, rods, monochromat

## Abstract

High-level visual functions such as reading and face recognition rely on global processes, which are often insensitive to high spatial frequencies. However, it is unknown whether a sharp cone signal is necessary for the development of these skills or whether a blurry rod signal is sufficient. CNGA3/B3-achromatopsia is a congenital disease stemming from cone dysfunction, leading to rod-only vision characterized by nystagmus, impaired acuity, and complete color blindness. We tested reading and face recognition in CNGA3/B3-achromatopsia patients (ACHM) to determine whether a rod signal is sufficient for these skills to develop. We tested 10 ACHM and 10 controls in three experiments under dark and light conditions. Initially, we evaluated acuity along the eccentricity axis. Later, we tested reading speed and upright/inverted face matching accuracy while tracking participants’ eye movements. Given that ACHM patients’ acuity under light conditions resembled that of controls under dark conditions, we selected these conditions for comparison. Remarkably, ACHM reading speed, face recognition abilities, and susceptibility to face inversion were not inferior to those of controls. Additionally, ACHM patients exhibited similar eye movements to controls, focusing their attention on specific areas of words and faces that indicate expertise. Despite the evident low-level limitations, ACHM patients demonstrated notable high-level visual skills, suggesting that rod-only vision is sufficient for the development of proficient reading and face recognition. These findings not only corroborate empirical evidence for high-level vision models but also enrich the discussion regarding the reasons for high-level deficits observed in individuals who have gained sight late in life.

## Introduction

Before the industrial era, humans engaged in high-level visual tasks even under low illumination conditions activating only rod photoreceptors (Lamb, 2016). Rod-mediated vision differs from cone-mediated vision in several ways. Spatially, rods are absent from the fovea (where cone density peaks), causing a foveal scotoma of around 1.5° of visual angle (Curcio and Allen, 1990; Curcio et al., 1990). In the periphery, scotopic acuity remains low due to extensive pooling of the rod signals (D’Zmura and Lennie, 1986). Additionally, rod-mediated vision is almost five times slower than cone-mediated vision (MacLeod, 1972). Third, unlike cone photoreceptors, rods have only one photoreceptor type, causing night vision to be colorless (Bowmaker and Dartnall, 1980). Overall, high-level visual performance might be compromised under scotopic conditions due to the foveal scotoma, reduced peripheral acuity and slower responses.

Recently, we were the first to study high-level visual behavior under scotopic conditions (McKyton et al., 2024). It is well-known that under photopic conditions, participants show a face inversion effect, demonstrating higher recognition performance for upright than inverted faces (Rhodes et al., 1993; Farah et al., 1995; Freire et al., 2000). Additionally, when viewing inverted faces under photopic conditions, participants perform more fixations overall and proportionally more fixations to the lower part of the face (nose, mouth) relative to upright faces (Barton et al., 2006). These two behaviors, considered evidence for the holistic perception of upright faces, were also observed under scotopic conditions. Similarly, reading under scotopic conditions was accurate, and participants executed a similar number of fixations to similar locations as under photopic conditions (McKyton et al., 2024).

To further understand the basis of this intact high-level performance under rod-only vision, we set out to test rod monochromats, patients who were born and whose visual system developed using rod vision alone. Complete achromatopsia, also known as rod monochromacy, is a rare cone dysfunction syndrome most commonly characterized by mutations in the CNGA3/B3 genes (Aboshiha et al., 2014). Patients (ACHM) have rod-mediated vision from birth and see the world in blurry shades of gray (Zobor et al., 2015). In addition to color blindness, the lack of functional cones causes impaired acuity and photoaversion (Johnson et al., 2004). Despite these limitations, ACHM can read, navigate, and recognize objects to the extent that they can lead independent lives. They do so even in daylight, probably since, unlike normally-sighted people, their rods do not bleach in high illuminations (Johnson et al., 2004), and photopic signals elicit activity in their early visual areas (Baseler et al., 2002; McKyton et al., 2021).

We set out to test two high-level visual tasks, reading and face recognition, in ACHM. We compared their results under light conditions to those of normally-sighted controls under scotopic conditions. This mixed-luminance comparison was used since it reduces noise stemming from the reduction in ACHM rod function under scotopic conditions (Baseler et al., 2002; Molz et al., 2023) (See Molz et al., 2023; “Relevance of mixed luminance comparison” for further explanation of this rationale). Under these mixed conditions, both groups had similar acuity and used rod input alone. Thus, the only relevant difference between the groups was the input used (rods/cones) during the development of the visual system.

Testing mid-level visual functions in ACHM showed that while global form perception might be impaired, global motion perception is not (Burton et al., 2016). However, little is known about ACHM high-level visual functions. Our first goal of this study was therefore to test ACHM high-level visual skills. If ACHM exhibit typical face inversion behavior, this would imply that they retain global face perception skills. If ACHM reading speed and fixation count do not exceed normally sighted parameters under dark conditions, this would suggest they are expert readers. For our second broader goal, we aimed to understand whether cone input is necessary for developing high-level perception. If the previously reported intact performance under rod-only conditions requires sharp cone input during visual development, we expect high-level visual deficits in rod monochromats.

## Methods

### Participants

Ten ACHM who manifested genetically confirmed CNGA3 or CNGB3 mutations were recruited at the Hadassah Medical Center (eight females; age 35.2 ± 10.7 years; Supplementary Table 1). All patients had nystagmus, photoaversion, complete color blindness (according to Farnsworth D-15) and visual acuity of 6/60 or lower, suggesting complete achromatopsia (Michaelides et al., 2004). All but one CNGB3 patient had a Full-Field Electroretinogram (FFERG) test with no detectible cone flicker response. CNGB3 mutations have not been linked with incomplete achromatopsia (Wissinger et al., 2001). Ten age-matched controls with normal vision were also recruited (eight females; age 32.6 ± 8.7). All participants spoke and read Hebrew as their first language. All participants completed a custom visual acuity test followed by the main experiments, which were each conducted in both light and dark conditions: Acuity along the eccentricity axis, Reading, and Face matching.

### Custom visual acuity test and adjusting individual light levels

Before the main experiments, acuity was tested binocularly under light and under dark conditions using a custom C chart presented on the same screens and distance used in the experiments. The custom chart was composed of six rows, each containing six black squared letters C (with leg width a third of the letter’s height and width) of the same size and at one of four orientations. The size of the C’s in each row was calculated such that the rows corresponded to visual acuity of 6/100, 6/60, 6/36, 6/24, 6/18, and 6/9. Under dark conditions, the white background luminance was within the scotopic range (0.002 cd/m^2^). Under light conditions, ACHM chose the white background luminance to minimize their perceived photoaversion, enabling them to achieve their maximal visual acuity. Minimizing photoaversion was also important for eye-tracking ACHM, who squint their eyes when exposed to high light levels. The light levels chosen for the light condition fell within the mesopic and photopic ranges (0.7–28 cd/m^2^). Participants’ acuity under light and dark conditions is shown in Supplementary Table 1.

### Experimental procedure

Experiments under light conditions were performed in a lit room. For ACHM, during the acuity and reading experiments, the screen showed black letters on each patient’s chosen white background luminance from the previous section. During the face matching experiment, the background was black, and ACHM chose the luminance level of the presented faces. Each control completed the experiments under light conditions at the luminance level chosen by their age-matched patient.

Experiments under dark conditions were performed in a dark room with a screen covered by neutral density filters (see below), and they were preceded by 20 min of dark adaptation.

The order of lighting conditions and the specific stimuli included in each lighting condition (faces and sentences) were counterbalanced across participants and conditions. However, all subjects began their session with the acuity experiment under light conditions, as this was used to familiarize subjects with the setup and screen.

### Eye tracking

All experiments were designed using Experiment Builder software (SR-Research). During the experiments, participants used a chin and headrest while an EyeLink 1000 eye tracker (SR-research) was used to collect monocular eye tracking data at 500 Hz. Each experiment started with an SR-research built-in five-point calibration procedure using a custom fixation point detectable also under a rod scotoma (see below, Fixation Point). However, due to patients’ nystagmus, a calibrated point could be inaccurate: instead of representing the averaged eye position across the nystagmus eye movement during fixation point presentation, it might represent the edge point in the nystagmus trace. Therefore, in each experiment, an additional custom calibration procedure was introduced. Five fixation points were presented sequentially (in the center, 11° left or right, and 7.8° above or below) for 4 s each, and eye position was recorded to allow recalibration of the space *post hoc* (see Supplementary material 1 and Supplementary Figure 1). This procedure was performed for both patients’ and controls’ eye movement data.

To create fixation maps, the default parameters of EyeLink Data Viewer were used (1° sigma; 300 ms window duration; 10% low activity cutoff).

### Screen

Stimuli were presented on a 1,280 × 1,024 pixel (37.5 cm × 30 cm) screen. Subjects sat 65 cm away from the screen, such that 39.8 pixels corresponded to 1° of visual angle. For experiments under light conditions, the screen had a luminance of 0.7–29 cd/m^2^ (according to ACHM choice) for white and 0.15 cd/m^2^ for black, measured using a Mavo-Monitor USB (Gossen) photometer. For experiments under dark conditions, a screen of the same size and resolution covered by three identical neutral density filters was used. Each filter reduced the screen’s luminance by a factor of 40 (according to our photometer measurements), resulting in a calculated screen luminance of ~0.002 cd/m^2^ for white and ~ 2E−06 cd/m^2^ for black (extrapolated).

### Fixation point

**Figure 1:**
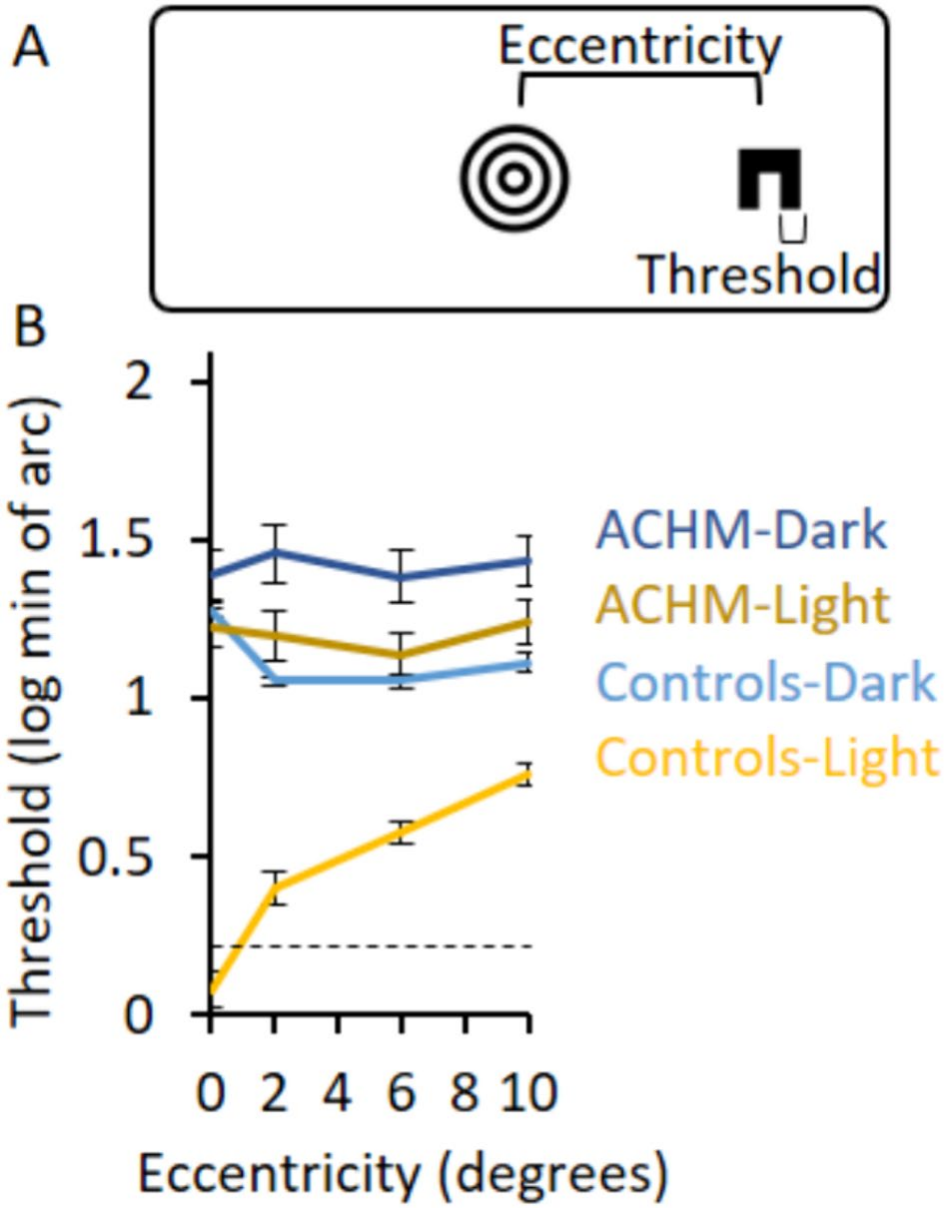
Acuity experimental design and results. **(A)** The experimental design shows the C letter presented at a certain eccentricity and the fixation point in the center of the screen. **(B)** The log of the acuity thresholds for ACHM and controls in the two lighting conditions as a function of eccentricity. Zero log threshold corresponds to 6/6 vision. Dashed lines in graphs represent the minimal threshold that could be measured directly; measurements below these lines are extrapolations (see Methods for details). Error bars represent ± SEM.

In all experiments, the fixation point was constructed from a set of black concentric circles (Figure 1A) that covered 3.75°. In this manner, the fixation point was visible under all conditions. When present, participants were instructed to fixate in its center.

### Acuity along the eccentricity axis

Subjects were presented with a central fixation point and instructed to press the spacebar. After 200 ms, a black letter C (with leg width a third of the letter’s height and width) was presented for 200 ms at one of four orientations (Figure 1A), randomly to the left or to the right of fixation. Subjects indicated the orientation of the C by pressing one of four keys.

Stimulus size (both letter size and leg width) was adjusted using a one-down one-up staircase procedure, with possible levels of 1.5, 3, 4.5, 6, 9, 13.5, 19.5, 28.5, 39, and 64.5 min of arc (for the width of one leg of the C). The staircase terminated after 10 reversals, with the average stimulus size across the last six reversals counted as the final acuity threshold. In the case of a ceiling effect, i.e., a participant answering the 1.5 min of arc (one pixel) stimulus correctly, a simulated reversal located on an incorrect trial for a 0.75 min of arc stimulus was added. This procedure served both to terminate the experiment even under a ceiling effect and to extrapolate the threshold to a value under 1.5 min of arc.

The staircase procedure was performed separately at eccentricities of 0, 2, 6, and 10°. If the C stimulus overlapped with the fixation point (at 0 and 2° eccentricities), a white disk slightly larger than the C stimulus served as the stimulus background so that the fixation point would not interfere with stimulus recognition. Otherwise, the entire fixation point was present during the whole trial.

Testing patient ACHM3 under dark conditions ended after a few trials since he/she could not perceive the stimulus even at its largest size.

### Reading

The reading experiment consisted of two phases: a learning phase and a test phase. Each trial began when participants fixated on a fixation point in the upper region of the screen above the area where the sentence was due to appear. After pressing a key, the fixation point disappeared, and a seven-word sentence in black Hebrew font, with a letter size of 1.2°, was presented in three lines (with two, three and two words in each line; Supplementary Figure 2A). The fourth word was of similar length and in the same screen position across all sentences. During the learning phase, subjects were presented with 10 sequential sentences and were directed to read each sentence before pressing a key to move to the next (reading speed was defined as the time between the two key presses). In the test phase, subjects were presented with 20 sequential sentences and were directed to press a key indicating whether each sentence was previously shown in the learning phase. Half of the sentences were shown previously. During the learning phase, reading speed and eye movements were recorded, and fixation maps were extracted from the eye-tracking evaluation. During the test phase, the number of correct responses was recorded.

Patient ACHM3 reported inability to read when presented with the example trial under dark conditions, leading to *N* = 9 for the ACHM group in the reading experiment under dark conditions.

### Face matching

Each trial began when participants fixated on a central fixation point and pressed a key. Afterwards, the fixation point disappeared, and subjects were presented with pairs of grayscale face images to the left and to the right of the previous fixation point. Subjects responded whether the two faces in the pair showed the same identity or not.

Face pair stimuli were taken from the Glasgow Face Matching Task 2 dataset (White et al., 2022). On the left side of each pair, a forward-facing, high-quality face image was presented (Supplementary Figure 3A), spanning about 22 × 14°. On the right side, a face of the same or different identity was presented, subject to alterations in head angle, pose, expression, or resolution. In all conditions, images were converted to grayscale and increased in contrast to 100% to make them easier to see in the dark, and the left face was adjusted such that its eyes appeared at the same position across images. To test for a face inversion effect, inverted face stimuli were generated by flipping the face pair images along the vertical axis.

In each lighting condition, subjects were shown a set of 50 upright and 50 inverted face pairs, with the order of upright and inverted blocks and the specific face pairs in each block counterbalanced across subjects. Face pairs were chosen such that each block had the same difficulty level (White et al., 2022) and half of the pairs shared the same identity. For a given subject, each face pair appeared only once across all experimental sessions. Correct responses as well as eye movements were recorded. Fixation maps were extracted from the eye-tracking recordings.

### Statistical analysis

Due to the small sample size, nonparametric unpaired Mann–Whitney or paired Wilcoxon tests were used for comparisons between or within groups, respectively.

### Eye-tracking analysis

#### Acuity along the eccentricity axis

Eye position data during stimulus presentation were extracted (see Supplementary Figure 4A for individual ACHM traces), and the standard deviation from fixation position was calculated as a measure of fixation stability.

#### Reading

To determine how many fixations a participant performed per word, we analyzed the second row of the learning phase sentences, comprising three words. This row was most suitable for eye tracking analysis since it was easy to pinpoint the start and the end of this phase of reading. Due to the ACHM nystagmus, we could not define “fixations” (nystagmus eye movements around a specific position) using standard parameters. Instead, for each participant (controls and ACHM) and trial, we manually identified the participant’s transition from the first to the second and from the second to the third row, and we manually counted fixations in the middle row as areas of clustered eye traces (Supplementary Figure 2). If, after moving to the third row, participants returned to the second row, these extra fixations were not counted. Three out of the 10 ACHM had severe nystagmus, such that “fixations” could not be distinguished from one another and could not be counted during the reading experiment, and thus we excluded their data. Supplementary Figure 2 shows participants’ eye movements and the attributed fixation counts during a learning phase trial.

#### Face recognition

To determine the area participants fixated during face recognition, we analyzed the eye-tracking data of all trials taken from the left side of the screen, excluding the right side and the 2° of the screen’s center (Supplementary Figure 3). The standard deviation of fixations’ Y positions was taken for analysis to compare between groups. One ACHM’s eye movements during the dark could not be measured due to low calibration quality.

## Results

### Acuity along the eccentricity axis

To test acuity at different eccentricities, participants identified the orientation of a letter C that appeared either to the left or to the right of a fixation point (Figure 1A) and changed size according to a staircase procedure. The acuity threshold was measured at varying eccentricities under light and dark conditions.

Achromatopsia acuity thresholds under light conditions were lower than under dark conditions (*p* = 0.005, 0.005, 0.005, 0.007; W = 0, 0, 0, 1 for eccentricities 0, 2, 6, and 10°, respectively) and higher than controls’ thresholds under light conditions (*p* < 0.0002; U = 0 for all eccentricities; Figure 1B). The two groups also differed in their acuity under dark conditions (*p* = 0.2, 0.0002, 0.0003, 0.0006; W = 33, 0, 2, 4 for eccentricities 0, 2, 6, and 10°, respectively). No difference was found between ACHM acuity thresholds under light conditions and controls’ thresholds under dark conditions (*p* = 0.10, 0.23, 0.67, 0.24; U = 28.0, 33.5, 44.0, 34.0; for eccentricities 0, 2, 6, and 10°, respectively).

Since deviation of the eye position from fixation creates noise in the accuracy of stimulus eccentricity, we calculated it as a measure of fixation stability. Standard deviation from fixation was 1.2° in the ACHM group (standard deviation: photopic horizontal 1.1 ± 0.6°; photopic vertical 1.3 ± 1.4°; scotopic horizontal 1.2 ± 0.3°; scotopic vertical 0.8 ± 0.5°) and 0.4° in the control group (standard deviation: photopic horizontal 0.5 ± 0.2°; photopic vertical 0.3 ± 0.1°; scotopic horizontal 0.5 ± 0.2°; scotopic vertical 0.4 ± 0.1°) across trials (Supplementary Figure 4A). While the inaccuracy in stimulus eccentricity presentation could affect the shape of the ACHM graphs in Figure 1A, the overall acuity level (showing that ACHM light condition results did not differ from controls’ dark condition results) should not be affected.

Consequently, we chose in the next sections to compare ACHM results under light conditions and controls’ results under dark conditions to measure ACHM high-level visual skills when tested under similar acuity limitations as controls. This is in addition to comparing the groups under dark conditions. Individual results are presented in Supplementary Figure 4B.

### Reading

To test reading performance and eye movement patterns, participants read 10 consecutive sentences in a learning phase that they were then asked to identify among 20 consecutive sentences in a test phase. Besides the success rate, which was determined by the test phase, all results were based on the learning phase, since participants were more likely to read the whole sentence in this section.

**Figure 2:**
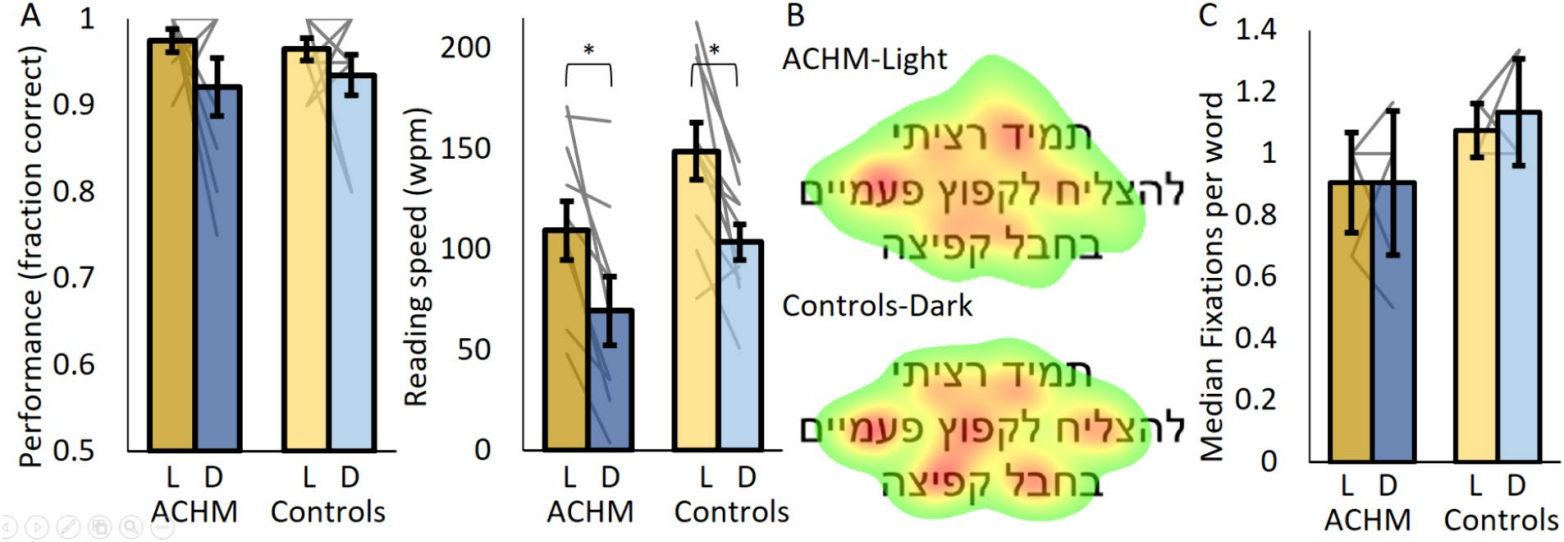
Reading performance and eye tracking results. **(A)** The fraction of correct responses and reading speed for ACHM and controls. **(B)** ACHM and controls’ average fixation maps across participants and sentences (normalized from green—fixated at 10% of the time, to red—most fixated). **(C)** The number of fixations per word for ACHM and controls. L, Light; D, Dark. Individual data points are connected using straight gray lines. *p < 0.05.

Both groups read slower under dark than under light conditions (ACHM: *p* = 0.005, W = 0; Controls: *p* = 0.009, W = 2; Figure 2A). When comparing ACHM results under light conditions and controls’ results under dark conditions, the fraction of correct responses (*p* = 0.19; U = 32.0) and reading speed (*p* = 0.73; U = 45.0) did not differ significantly. Similarly, no significant difference was found between groups under dark conditions (fraction of correct responses: *p* = 0.9; U = 42.5; reading speed: *p* = 0.078; U = 23.0).

Despite ACHM nystagmus, we managed to record “fixations” (typical nystagmus eye movements around a specific position) in seven out of 10 ACHM. ACHM performed “fixations” to similar locations in the presented sentence as controls (Figure 2B; Supplementary Figure 2B). Additionally, the median number of “fixations” per word in the middle row of the screen did not differ significantly between ACHM under dark and controls under light conditions, with even a slight trend suggesting lower fixation number in the ACHM (*p* = 0.058; U = 15; Figure 2C). Similar results were obtained when comparing both groups under dark conditions (*p* = 0.11; U = 18).

### Face matching

To test for face recognition, participants were asked whether two faces shown on both sides of the screen shared the same identity or not. The left faces in all pairs faced forward and were aligned to the same location, and they were therefore used for eye tracking analysis. The test was administered with blocks of upright and inverted faces under light and dark conditions.

**Figure 3:**
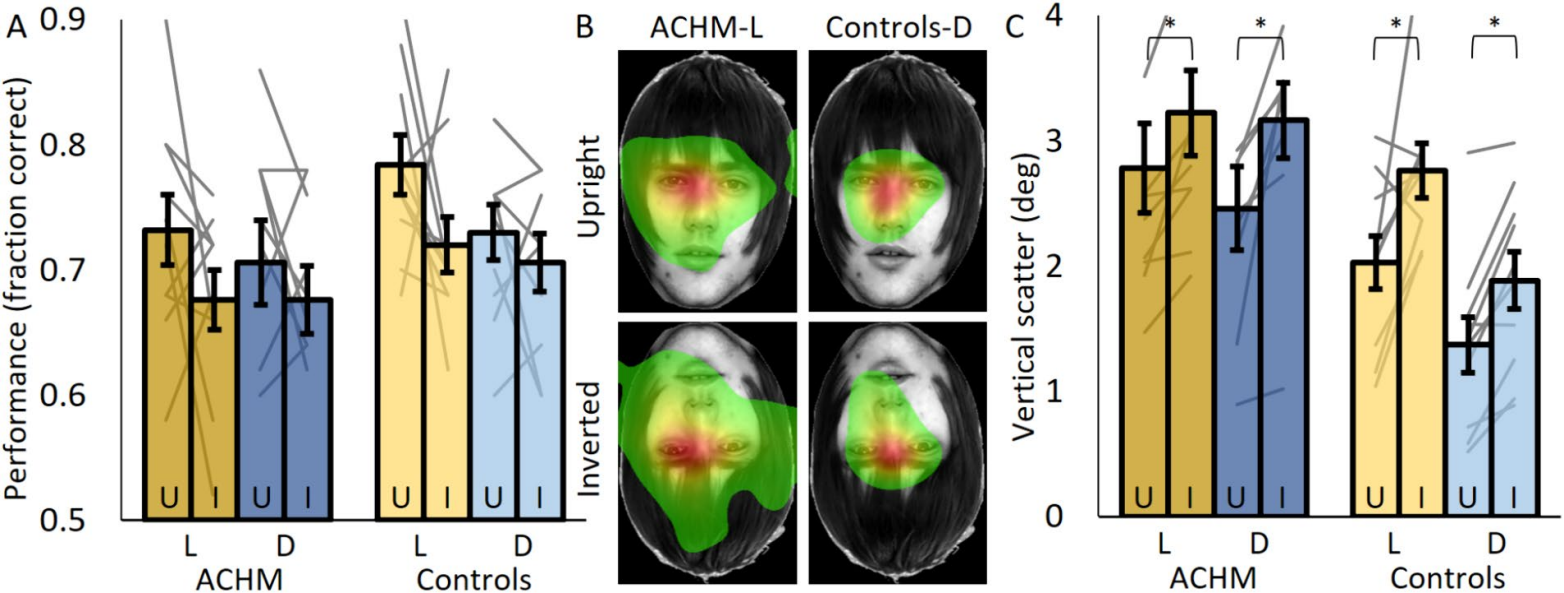
Face recognition performance and eye tracking results. **(A)** The fraction of correct responses for upright (U) and inverted (I) faces for ACHM and controls. **(B)** ACHM and controls’ average fixation maps across participants (normalized from green—fixated 10% of the time, to red—most fixated). **(C)** The vertical scatter of eye movements defined by the standard deviation of eye position across the *Y* axis. L, Light; D, Dark. Individual data points are connected using straight gray lines. Face images were taken from The Glasgow Face Matching Task dataset (White et al., 2022). *p < 0.05.

Generally, both groups performed above chance under all conditions (see Supplementary material 2 for analysis of individual subjects’ face recognition results). No significant difference between controls under dark conditions and ACHM under light conditions was evident for both upright (*p* = 0.88; U = 23) and inverted (*p* = 0.35; U = 37) faces (Figure 3A). This was also true when comparing both groups under dark conditions (upright: *p* = 0.82, U = 46.5; inverted: *p* = 0.52, U = 41). Performance for upright faces was higher than for inverted faces, but this difference was not significant for both patients (light: *p* = 0.24; W = 16, dark: *N* too small for *p*, W = 10) and controls (dark: *p* = 0.31, W = 17.5; light: *p* = 0.08, W = 10.5). At the individual level, under light conditions, only two ACHM (*p* = 0.003; 0.04) and three controls (*p* = 0.03; 0.01; 0.009) showed a significant face inversion effect (Supplementary material 2).

Fixation maps showed that for both groups and face orientations, most fixations were executed to similar locations around the eyes region (Figure 3B; Supplementary Figure 3). ACHM eye movements under light or dark conditions covered a larger area of the face than controls’ under dark conditions (expected due to nystagmus; comparing vertical scatter between controls’ dark and ACHM light conditions for upright: *p* = 0.003; U = 10; for inverted: *p* = 0.007; U = 14; comparing vertical scatter between controls’ dark and ACHM dark conditions for upright: *p* = 0.02; U = 17; for inverted: *p* = 0.004; U = 9). However, for both groups, the area covered by fixations increased when the faces were inverted. Vertical scatter (represented by the standard deviation of the fixation positions; Figure 3C) was larger for inverted faces for both controls (*p* = 0.007; W = 1) and patients (Light: *p* = 0.005; W = 0; Dark: *N* too small for *p*; W = 2). At the individual level, under light conditions, 9/10 ACHM and 8/10 controls had a significant effect showing larger variance when viewing inverted faces (*p* < 0.001) (Supplementary material 2).

## Discussion

Achromatopsic individuals exposed solely to rod input from birth demonstrated comparable high-level visual performance and similar scanning strategies to sighted people under scotopic conditions. We propose that, beyond the intriguing clinical question into how this unique ACHM cohort reads and recognizes faces, our work may have implications for understanding visual development under degraded input.

The group we tested, although not large, corresponds in terms of its visual acuity characteristics to previous reports in the literature. Similar to previous findings, our ACHM cohort exhibited lower visual acuity compared to controls, both under light and dark conditions (Sloan and Feoick, 1972). Furthermore, patients’ visual acuity ranged between 6/60 and 6/100, similar to Nishiguchi et al. (2005) reports of visual acuity in the range of 20/200 to 20/400 for CNGA3 and CNGB3 mutations.

At such low acuity levels, inability to solve high-level visual tasks might be due to lack of relevant informative input as opposed to lack of high-level visual function development. Furthermore, these tasks might necessitate the cones’ high-temporal processing. However, our previous paper investigating reading and face recognition under scotopic conditions (in the normally sighted) suggests that rods and low acuity are sufficient for solving these tasks as long as the stimuli are large enough (McKyton et al., 2024).

Indeed, despite these low-level limitations, ACHM showed preserved high-level visual perception skills. Regarding face perception, ACHM upright face recognition did not differ from controls’, and ACHM showed a trend of a face inversion effect, suggesting global face perception. Since controls’ results were similar, the inability to reach a significant face inversion effect probably resulted from the small sample size. Regarding eye movements during face recognition, patients differed from controls in having larger fixation areas for both upright and inverted faces, as expected due to their nystagmus. However, like controls, they showed a significant increase in their fixation scatter when faces were inverted, another indication of global face perception. The larger impact of face inversion on eye movements rather than on correct responses was observed in the individual-level statistics for both ACHM and controls, suggesting similarity in face recognition mechanisms between the groups. As for reading, ACHM reading speed and fixation number in the light did not differ from controls’. More fixations could have indicated local instead of global reading, which is linked with reading difficulties (Franceschini et al., 2017).

Notably, proving that two groups are alike is problematic, and insignificant comparisons between ACHM and controls’ performance could theoretically stem from our small sample size. However, ACHM performance under light conditions was generally (though not significantly) higher than controls’ under dark conditions, even though their average acuity was lower. On average, not significantly, ACHM recognized upright faces better than controls, showed a larger face inversion effect, and read faster, more accurately and with fewer fixations per word. These results suggest that ACHM high-level visual performance is probably not worse than controls’ performance under rod-only conditions.

Some theories suggest that high-level visual functions remain intact under low-acuity visual development. Vogelsang et al. (2018) suggested that the poor acuity of healthy infants might be advantageous for early face concept learning. They claimed that high-resolution inputs could even impair face recognition, leading subjects to focus on fragmentary data rather than capturing the gestalt of faces. Here we show empirical evidence supporting these theories, demonstrating that the blurry rod signal ACHM use from birth is sufficient for face concept learning and the development of reading skills.

This empirical evidence is also relevant for research in the newly sighted. Newly sighted individuals who were congenitally blind and to whom vision was restored at a late age have low acuity levels (Ganesh et al., 2014) and deficits in high-level vision (Le Grand et al., 2004; Ostrovsky et al., 2006; McKyton et al., 2018; Zohary et al., 2022), even years after restoration. Theoretically, their high-level deficits could stem either from their current impaired low-level input or from lack of input during a critical period. Since we found that high-level vision can still develop under low acuity levels, our results support the latter hypothesis.

In conclusion, our study quantifies ACHM high-level visual abilities and shows that these abilities do not fall short of controls’ under scotopic conditions. Beyond that, the study implies that a rod-only signal is sufficient for the development of high-level visual skills and provides valuable insights into the development of high-level vision under blurred input.

## Data Availability

The raw data supporting the conclusions of this article will be made available by the authors, without undue reservation.
