# Supplementary material for: Intact high-level visual functions in congenital rod-monochromacy
Source: Front Neurosci. 2024 Sep 27;18:1418916. doi: 10.3389/fnins.2024.1418916 (PMC11466936; doi:10.3389/fnins.2024.1418916)
Supplement: Supplementary file 1 [file Data_Sheet_1.pdf]

Supplementary Data

Patient #	Age (y)	Gender	Genotypes	Visual Acuity Under Light (Matched Control)	Background Luminance (cd/m^2)	Visual Acuity Under Dark (Matched Control)	Cone Flicker FFERG Response
1	45	F	CNGA3	6/60 (6/18)	1.7	6/100 (6/60)	ND
2	43	F	CNGA3	6/100 (6/18)	18	6/100 (6/60)	ND
3	24	F	CNGA3	6/100 (6/9)	28	6/100 (6/24)	ND
4	18	F	CNGB3	6/60 (6/9)	1.7	6/100 (6/36)	NA
5	35	F	CNGB3	6/60 (6/18)	0.7	6/100 (6/60)	ND
6	48	F	CNGA3	6/60 (6/18)	3	6/100 (6/60)	ND
7	23	M	CNGB3	6/100 (6/18)	1.7	6/100 (6/60)	ND
8	43	F	CNGB3	6/60 (6/18)	0.7	6/60 (6/60)	ND
9	30	F	CNGB3	6/100 (6/18)	1.7	6/100 (6/36)	ND
10	43	M	CNGA3	6/60 (6/9)	14	6/100 (6/100)	ND

Supplementary Table 1. Demographic and clinical details of the 10 ACHM with established genotypes. The background luminance is of the white background chosen during the custom visual acuity test, and this was presented during the Acuity along the eccentricity axis and the Reading experiments under light conditions. FFERG results for cone flicker response: ND – non-detectable; NA – not available.

Supplementary Text 1 – Custom calibration analysis

From each fixation point location (5 locations total, Supplementary Figure 1A; crosses), data were collected from the last 3500ms out of the 4000ms of fixation point presentation (Supplementary Figure 1A&B; yellow traces), and the median eye position in each location was calculated (Supplementary Figure 1A; circles). A linear transformation was then executed to calibrate the eye position in space. First, data were aligned such that the eye position during presentation of the central fixation point corresponded to the central fixation location. Later, to calibrate the left visual space, data were linearly transformed such that data collected during the left fixation point presentation corresponded to the X position of the left fixation point. Data were transformed similarly for the right, upper and lower spaces (Supplementary Figure 1B).

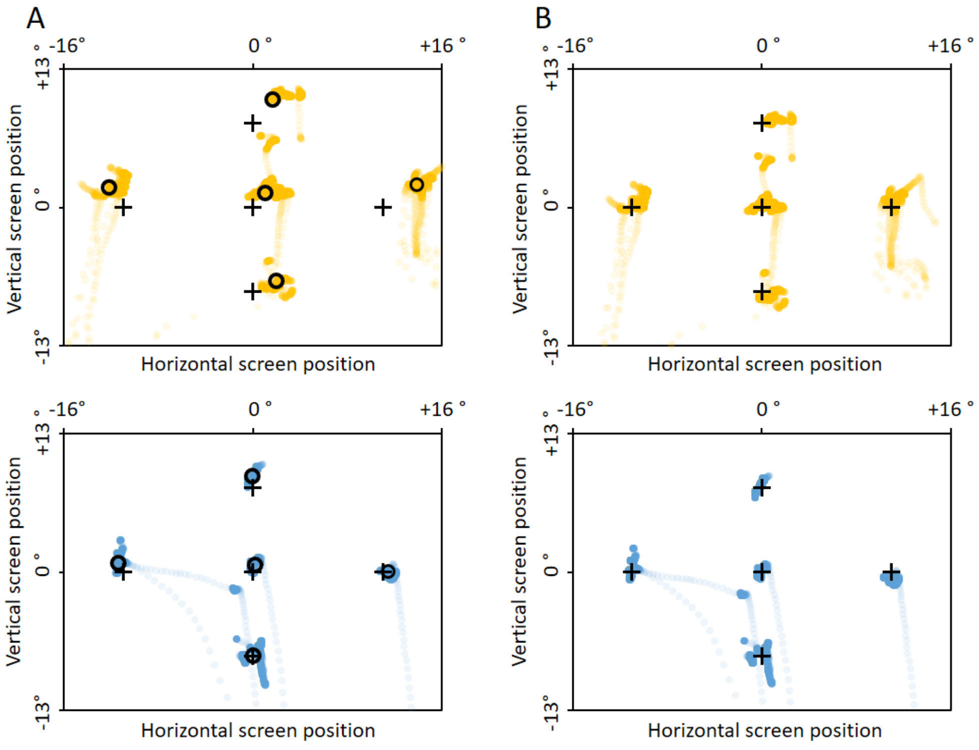

Supplementary Figure 1. Custom calibration procedure.

(**A**) Eye positions collected during the custom calibration procedure for one patient under light conditions (yellow) and his/her matched control under dark conditions (blue). Black crosses represent the fixation point positions, and circles represent the median eye positions for each fixation point position. (**B**) Eye positions of the same participants after post-hoc linear calibration.

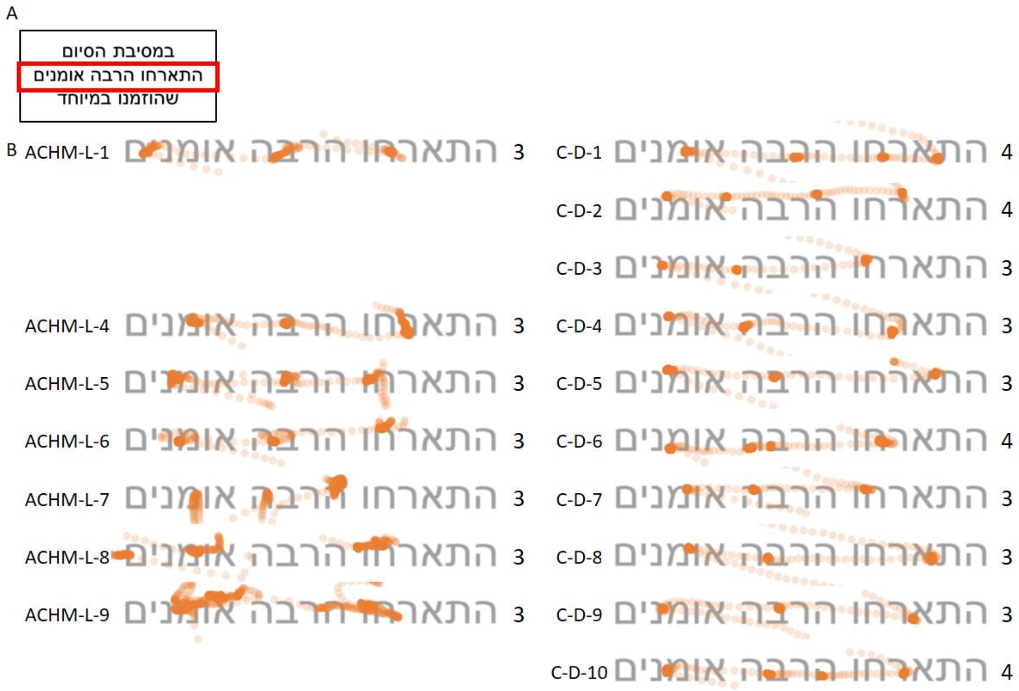

Supplementary Figure 2. Reading experiment individual eye tracking results.

(**A**) An example sentence stimulus. In each sentence, the middle line, containing 3 words, was used for eye tracking analysis. (**B**) One trial for each participant from the learning phase, representing typical eye tracking results for that participant. For each participant, the number represents the number of fixations that were determined for that trial. Three ACHM (2, 3, 10) had such a severe nystagmus that fixations could not be distinguished or localized to a certain line in the sentence. C-controls; L-light; D-dark.

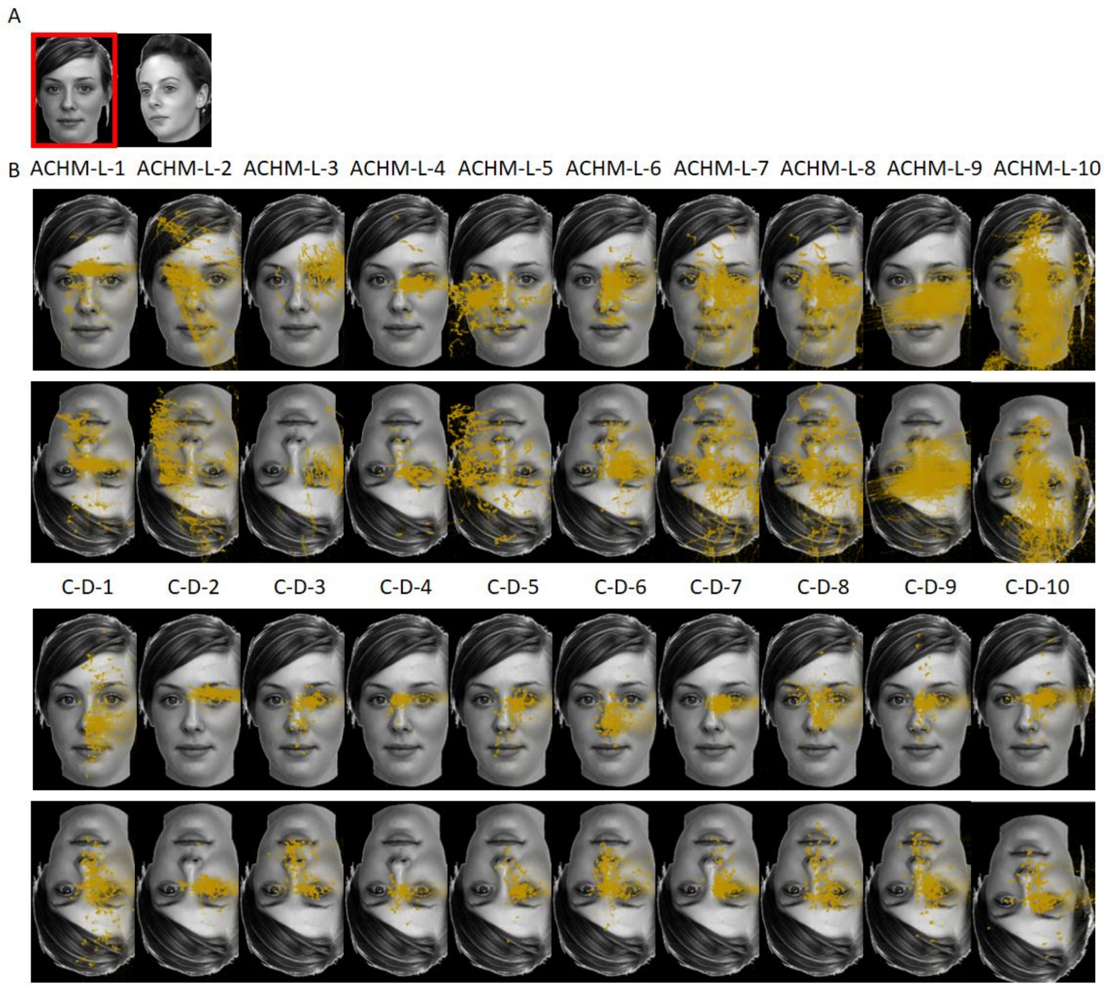

Supplementary figure 3. Face recognition experiment individual eye tracking results.

(**A**) An example face pair stimulus. For each face pair, the left face, which was always facing forward and in the same position, was used for eye tracking analysis. (**B**) For each participant, fixations across all trials for upright and inverted faces. C-controls; L-light; D-dark.

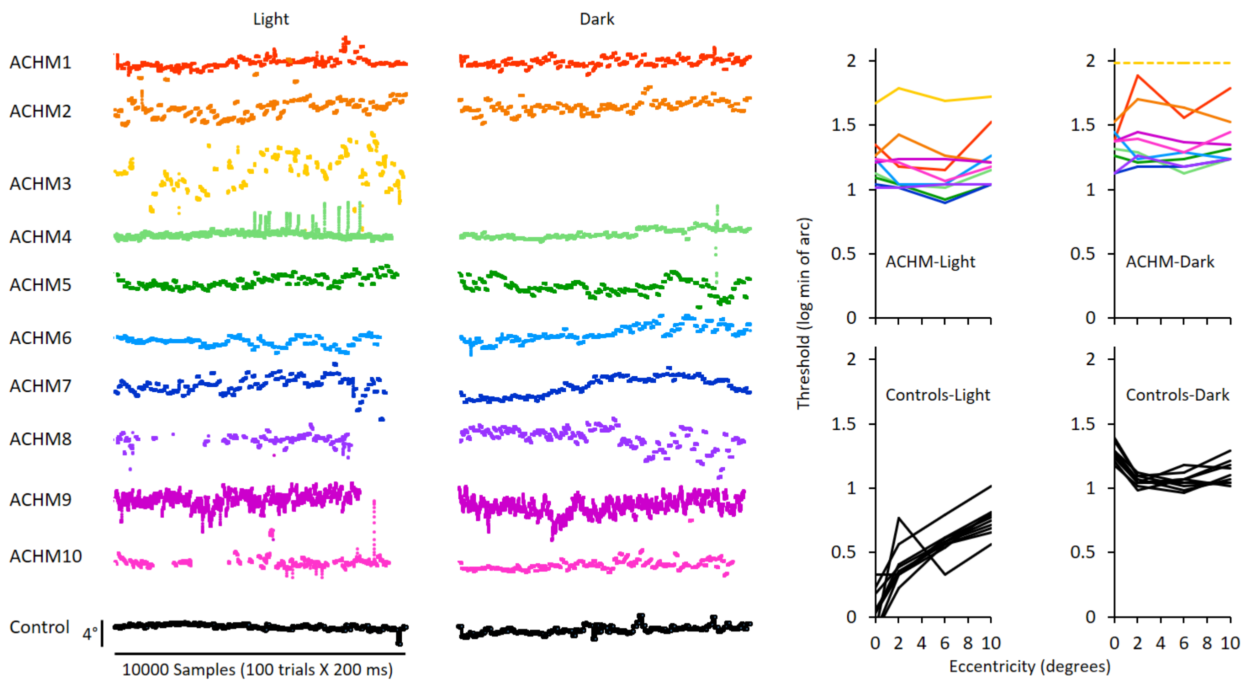

Supplementary Figure 4. Individual fixation stability and acuity results.

(**A**) For each ACHM, horizontal eye movements from 100 trials (200 ms each) during the stimulus presentation in the acuity experiment. (**B**) The log of the acuity thresholds for ACHM and controls in the two lighting conditions as a function of eccentricity. Each color represents one ACHM. Dashed line shows floor effect for ACHM3.

Supplementary Text 2 – Individual face recognition statistics

To explore individual participants' face recognition skills, we first tested whether each participant could recognize faces above chance level (face recognition effect). We performed binomial testing on each participant's correct/incorrect responses in both light and dark conditions and in upright and inverted face presentations.

Under light conditions, 9/10 ACHM and 10/10 controls had a significant face recognition effect for upright faces ($p<.05$). One ACHM had a marginal face recognition effect ($p=.06$). For inverted faces, 8/10 ACHM and 9/10 controls had a significant face recognition effect. One ACHM had a marginal face recognition effect ($p=.06$), and one ACHM had no effect ($p=.1$)

Under dark conditions, all participants had a face recognition effect for both upright and inverted faces.

Next, using t-tests, we tested whether each participant showed a significant face inversion effect.

Under light conditions, 7/10 ACHM and 8/10 controls showed better recognition for upright than for inverted faces, but only two ACHM ($p=.003$; .04) and three controls ($p=.03$; .01; .009) had a significant face inversion effect. One control had a significant effect in the other direction, with better recognition for inverted than upright faces ($p=.03$).

Under dark conditions, 5/10 ACHM and 6/10 controls showed better recognition for upright than for inverted faces, but none of the participants had a significant face inversion effect.

Lastly, we used an F test to test whether the variance of the eye position on the y-axis was statistically different between upright and inverted faces.

Under light conditions, 9/10 ACHM and 8/10 controls had a significant effect showing larger variance when viewing inverted faces ($p<.001$). One ACHM and two controls had a significant effect in the other direction ($p<.001$).

Under dark conditions, 8/9 ACHM and 9/10 controls had a significant effect showing larger variance for viewing inverted faces ($p<.008$). One ACHM had a significant effect to the other direction ($p<.001$), and one control's results were not significant ($p=.30$).
